# AI-Generated “Slop” in Online Biomedical Science Educational Videos: Mixed Methods Study of Prevalence, Characteristics, and Hazards to Learners and Teachers

**DOI:** 10.2196/80084

**Published:** 2025-11-20

**Authors:** Eric M Jones, Jane D Newman, Boyun Kim, Emily J Fogle

**Affiliations:** 1 Department of Foundational Medical Studies, Oakland University William Beaumont School of Medicine, 586 Pioneer Drive, Rochester, MI, 48309, United States, 1 248-370-3731; 2 Department of Human Development and Child Studies, Oakland University, Rochester, MI, United States; 3 Department of Chemistry and Biochemistry, California Polytechnic State University, San Luis Obispo, CA, United States

**Keywords:** generative AI, artificial intelligence, YouTube, TikTok, biochemistry education, medical biochemistry, cell biology education, basic medical sciences education, medical education, slop, careless speech

## Abstract

**Background:**

Video-sharing sites such as YouTube (Google) and TikTok (ByteDance) have become indispensable resources for learners and educators. The recent growth in generative artificial intelligence (AI) tools, however, has resulted in low-quality, AI-generated material (commonly called “slop”) cluttering these platforms and competing with authoritative educational materials. The extent to which slop has polluted science education video content is unknown, as are the specific hazards to learning from purportedly educational videos made by AI without the use of human discretion.

**Objective:**

This study aimed to advance a formal definition of slop (based on the recent theoretical construct of “careless speech”), to identify its qualitative characteristics that may be problematic for learners, and to gauge its prevalence among preclinical biomedical science (medical biochemistry and cell biology) videos on YouTube and TikTok. We also examined whether any quantitative features of video metadata correlate with the presence of slop.

**Methods:**

An automated search of publicly available YouTube and TikTok videos related to 10 search terms was conducted in February and March 2025. After exclusion of duplicates, off-topic, and non-English results, videos were screened, and those suggestive of AI were flagged. The flagged videos were subject to a 2-stage qualitative content analysis to identify and code problematic features before an assignment of “slop” was made. Quantitative viewership data on all videos in the study were scraped using automated tools and compared between slop videos and the overall population.

**Results:**

We define “slop” according to the degree of human care in production. Of 1082 videos screened (814 YouTube, 268 TikTok), 57 (5.3%) were deemed probably AI-generated and low-quality. From qualitative analysis of these and 6 additional AI-generated videos, we identified 16 codes for problematic aspects of the videos as related to their format or contents. These codes were then mapped to the 7 characteristics of careless speech identified earlier. Analysis of view, like, and comment rates revealed no significant difference between slop videos and the overall population.

**Conclusions:**

We find slop to be not especially prevalent on YouTube and TikTok at this time. These videos have comparable viewership statistics to the overall population, although the small dataset suggests this finding should be interpreted with caution. From the slop videos that were identified, several features inconsistent with best practices in multimedia instruction were defined. Our findings should inform learners seeking to avoid low-quality material on video-sharing sites and suggest pitfalls for instructors to avoid when making high-quality educational materials with generative AI.

## Introduction

### Background

Video-sharing platforms such as YouTube (Google) and TikTok (ByteDance) have become entrenched features of the educational landscape. Both instructors and students rely on these resources for a variety of purposes relating to teaching and learning [1-3], and the inherent benefits of video instruction in science education, specifically, have been well-documented [4-6]. Nonetheless, these video-sharing platforms’ greatest advantages—accessibility and low barriers to creating and sharing—are also arguably their greatest weaknesses, as the lack of barriers leads naturally to large amounts of low-quality material appearing alongside authoritative, high-value material. Because these platforms rely mostly on advertising for revenue, algorithms that recommend videos based on past views [7] and prioritize engaging over reliable videos [8] make it difficult to find the most credible and useful videos, not simply those most likely to maximize time on the site [8]. Furthermore, not all audiences may have the motivation or ability to assess the reliability of online videos [9,10], and in the absence of a shared standard of quality, one audience might find a video informative while another finds it inappropriate. Frameworks for assessing the quality of multimedia instruction have been advanced over the past few decades [6,11-13], but these are directed toward educators and instructional designers. How learners judge the quality of educational content remains poorly studied, and in the absence of guidance, students may simply defer to intuition [14].

The accessibility of social media platforms, and the lack of any uniform standard for judging quality, inherently present a challenge to any learner searching for educational content. The recent explosion in generative artificial intelligence (genAI) technologies, including large language models (LLMs) like ChatGPT (OpenAI) and Claude (Anthropic), and image generators like Stable Diffusion (Stable Diffusion AI) and Midjourney [15], has added further complication to this situation. To genAI users, time and effort are no longer barriers to generating shareable content. As a result, writing websites [16], social networks [17,18], and online markets [19] are increasingly cluttered with fake artificial intelligence (AI)–generated essays, posts, artwork, and merchandise. More troublingly, the scientific literature is now polluted with false machine-generated studies and data [20,21], often to push contrarian agendas [22]. The low-quality, high-volume, AI-generated content behind these examples has been called “slop” [19]; its proliferation led *MIT Technology Review* to deem slop the “biggest AI flop” of 2024 [23]. Slop lacks a single widely accepted definition, but journalists and industry commentators generally agree that slop is of low quality, ubiquitous, lacking in artistic or scientific value, and generated to maximize exposure or engagement, or simply to fill space on sharing platforms [16,19,24]. At best, slop is a distraction requiring time and effort to sift through in search of good material; at worst, it presents specific dangers to learners, educators, creative professions, and the overall atmosphere of public information [24].

The degree to which slop has crept into educational materials is largely unknown. Exploratory studies in medical and undergraduate science education suggest that carelessly produced genAI content can pose a significant risk of misunderstanding, spread outright misinformation [25,26], or promote deskilling or “metacognitive laziness” [25,27] in learners. Learners turn to AI content when they are most easily influenced—while uncertain or confused—and thus are most likely to be persuaded by errors or biases in the genAI output [28]. Yet errors abound in such content; in medical teaching, genAI output has been found to misrepresent rare diseases or those with variable presentations [29,30], and AI-generated anatomical diagrams often contain gross inaccuracies [31,32]. In other fields, genAI has been shown to create garbled chemical models and biased depictions of researchers [33,34], inaccurate but plausible-sounding descriptions of metabolic processes [35], and realistic images of nonexistent animal species, leading to confusion in biodiversity conservation efforts [36].

As for video, it is possible to create entirely AI-generated video clips using tools such as Synthesia and Sora (OpenAI); however, at the time of writing, these tools (unlike those above) are not yet available for free use, or free users are limited to very short clips. Consequently, fully AI-generated videos are not yet as widespread as images and text. This does not, however, mean video-sharing sites are free of slop. Free users can, for example, use AI tools to animate a photo of a “narrator,” or stitch together stock or AI-generated images to create a longer video. Tutorials on making videos in this fashion are widely available [19,37].

The purpose of this study is to examine the reach and characteristics of lazily-made genAI content in online videos on preclinical biomedical sciences (medical biochemistry and cell biology; eg, Biochemistry & Nutrition and Cell Biology & Histology topics in the USMLE Foundational Sciences area, as these are the authors’ disciplines of expertise). Although slop has been widely discussed in popular media, it has not yet received much scholarly attention, so our first priority is to establish a useful definition of slop in educational media. To this end, we use the theoretical framework of “careless speech” recently advanced [38] to propose legal-ethical responsibilities of genAI.

### Theoretical Framework

The characteristics of slop are ultimately a consequence of genAI’s intrinsic structure. All present genAI tools work by predicting associations between linguistic elements, based on human-reinforced training with real (human-generated) data. They cannot directly access external reality. They may therefore state falsehoods as fact or realistically depict impossible scenes (so-called hallucinations or confabulations), so long as the output correlates with training data [38]. GenAI is also prone to subtle errors or omissions in addition to outright falsehoods; it has difficulty grasping humor, nuance, or insinuation [26]; it does not exhibit a clear concept of uncertainty and tends to make confident assertions even where there is no clear answer [38,39]. Most genAI tools are programmed to sound authoritative and to give responses deferentially and in accord with a user’s desires (sycophancy) [40]. For any AI-generated task, biased or incomplete information in the training data (eg, absence of an important but uncommon viewpoint) will result in biased output [41,42]. The “speech” generated by AI (which we mean here to encompass not only language but also images, video, sounds, and other output), therefore, appears authoritative and competent but is unmoored from physical reality, lacks any motivations or principles, and carries whatever biases are present in the training data.

Can AI-generated speech be trusted, then? A recent paper by Wachter and colleagues [38] suggests the answer is no, unless there is cross-validation with the outside world, for example, using “human in the loop” [43,44] or “zero-shot translation” [45] approaches to verify accuracy, forestall errors, and account for uncertainty or caveats in the output. Wachter and colleagues use the term “careless speech” to describe the type of output that unsupervised AI produces. Careless speech is quasi-factual output that correlates with what humans say is reality (the training data), but without direct access to that reality; it is a coherent statement approximating a factual statement. Careless speech is not necessarily misinformation, as it is not always false. Rather, it is independent of reality; it may resemble truth, but exists separately.

We believe careless speech is a useful framework for establishing a definition of slop relevant to educational material. From this point onward, we use the term “material” to refer to a specific created object (eg, a video), and reserve the word “content” for the subject matter (contents) of these materials.

We define slop as follows: slop is any material, created mostly or entirely by generative AI, with little or no apparent human care toward the accuracy, fluency, or helpfulness of the material or of its most likely use or interpretation.

The operative word in this definition is *care*, to which we assign two meanings [46]: (1) deliberate attention and effort (eg, prompt design, editing, and fact-checking) toward ensuring the material has desirable characteristics, that is, to “care for” (taking on “responsibility to meet a need that has been identified” [46]); and (2) some professional or personal stake in the outcome, implying an ownership of and accountability for the product and its likely uses, that is, to “care about.” AI-generated material that does not discernibly exhibit care in *both* senses of the term is slop, regardless of its accuracy. Our definition implies that any material made entirely by genAI is slop; material made by genAI with human intervention may or may not be slop, depending on the degree of care in the intervention.

Our definition makes no direct reference to the quality of the material. This is intentional. If one defines “quality” in terms of accuracy and realism, genAI is making tremendous strides in improving its quality. Yet just as the content (meaning here the messaging and subject matter) of AI output is, at best, incidentally accurate—in Wachter’s words, “True responses are an accident of probability and reinforcement via human feedback, not agency or a conception of truth or intent to tell the truth” [38]—the quality of AI output is, at best, incidentally good. Without a caring human in the loop, AI output can only approximate, by correlation, characteristics associated with quality. Thus, slop is independent of quality in the same way that careless speech is independent of reality.

Likewise, our definition is agnostic regarding the purpose for which the AI-generated material is made. Slop is often made and disseminated to game engagement metrics (eg, clickthroughs, likes, and views), ultimately for the creator’s financial or political gain [19,24]. However, it is possible that some slop is made with the genuine intent of informing or entertaining—only without adequate care to ensure this intent is fulfilled. We therefore believe intent and purpose are irrelevant to the definition of slop, particularly since the intentions of those generating the slop are generally unknown.

### Educational Implications of Slop and Careless Speech

Careless speech was proposed as a framework for understanding the dangers of genAI output in legal settings. In educational settings, genAI is likely to present a distinct set of risks. Much of the research on educational hazards of genAI focuses narrowly on non-factual or hallucinated output [30-32,35,36] or on bias and ethical risks [34,47-50]. A more holistic understanding of the impact of careless speech on learning requires a broad framework for what makes educational materials effective at all.

With respect to video, the theory of multimedia learning [51] is one such framework and is supported by considerable empirical evidence [4]. Drawing from cognitive load theory, this model considers structural and design features that influence the effectiveness of multimedia materials. Complementary to this theory, other models emphasize the content of multimedia materials, specifically themes of active learning and features that promote engagement with the video [6,12,13]. Thus, the effectiveness of multimedia educational tools may be seen as having both design or structural components and content components that support learning.

Because the concern of this study is the educational effectiveness of video, some might question our choice to focus on AI slop rather than low-quality video more generally. There are at least 2 reasons why slop deserves particular attention. First, the sheer volume of slop is already overwhelming online platforms [16,18,19]. Students are thus extremely likely to encounter slop, which may soon comprise the majority of low-quality video. Second, instructors wishing to use genAI responsibly need to know the likely failure modes of the technology, so that proper attention can be paid to avoiding these pitfalls, maximizing educational impact, and reinforcing the necessity of human judgment in human-centered professions [52].

This study examines the current prevalence and problematic characteristics of slop educational videos on popular platforms, according to the following three research questions (RQs):

- RQ1: What are the qualitative characteristics of slop videos that might imperil learning or trust in educational systems?
- RQ2: What is the prevalence and reach of slop, according to our definition, in medical biochemistry or cell biology material on YouTube and TikTok, as discerned from viewership data?
- RQ3: Are there any quantitative metrics, such as view or like rates, that can reliably identify slop videos?

To address RQ1, we rely on a 2-stage qualitative content analysis in which we identify the educationally hazardous traits of likely slop videos and map them to the 7 characteristics of careless speech identified by Wachter et al [38]. We approach RQ2 and RQ3 using basic data mining methods. We hypothesize that slop videos display features contravening both the structural and content-thematic elements of effective multimedia instruction and present a significant (and growing) share of the educational space on these platforms.

## Methods

### Study Design and Approach

A complete description of the search and screening procedure is given in Multimedia Appendix 1. The overall strategy was to search YouTube and TikTok for videos on biochemical topics that first-year medical students often find challenging, to examine these videos for signs of careless AI use, and to compile a list of problematic features of the suspected genAI videos (RQ1). Data on viewership, video age, and duration were also collected to infer the reach and popularity of these videos, compared with the entire dataset (RQ2), and to see if these correlated with slop (RQ3).

### Searching and Screening of Videos

YouTube and TikTok were searched using third-party application programming interfaces (APIs; SerpAPI YouTube Search Engine and Apify TikTok Search API) over a 2-week period in late February and early March 2025. In total, 10 queries were used for each platform (Table 1), incorporating both single-word and sentence-like queries to obtain a variety of results.

**Table 1.** Summary of search results for each of the 10 queries. Unique URLs include off-topic and non-English videos that were excluded at later stages of screening.

Search query	YouTube URLs (raw), n	YouTube URLs (unique), n	TikTok URLs (raw), n	TikTok URLs (unique), n
How do enzymes work	209	114	60	—^a^
Protein secondary versus tertiary structure	210	96	60	—
Ion channel function	154	66	89	—
Cell cycle regulation	198	105	60	—
Carbohydrate metabolism	59	51	60	—
Electron transport chain	208	101	84	—
Urea cycle	68	67	42	—
Pentose phosphate pathway	195	109	60	—
Cytoskeleton	185	114	60	—
What are eicosanoids	110	85	88	—
Total	1596	908	663	617

a Not available, as the TikTok URLs were deduplicated as a single group.

Residential proxy servers were used, and stored browser data were cleared before each search. The search results were exported in JSON format, from which bare URLs were extracted and deduplicated, resulting in 908 and 617 unique video links for YouTube and TikTok, respectively. These videos were viewed for a few seconds each to identify off-topic and non-English videos, which were excluded. The 1082 on-topic videos remaining (814 YouTube and 268 TikTok) were then screened briefly for common “tells” of AI-generated material (refer to Multimedia Appendix 1 for rubric); 1 screener (EMJ) viewed each video for a minimum of 30 seconds (or the whole video, if less than 30 s) and flagged any showing signs suggestive of genAI. A list of these videos was then distributed among 3 reviewers, who viewed them in their entirety to verify that the “tells” were present and to provide a score (0=not AI-generated, 1=partially AI-generated, and 2=mostly AI-generated), and to note any factual errors. These videos were labeled as “likely AI-generated” (but not necessarily slop) if the average score was 1.0 or higher (only 1 video did not meet this criterion). AI detection tools were not used, due to their known unreliability [53].

To determine viewership, video metadata (including days online, duration, number of views, likes, and comments) was scraped for the entire 1082-video dataset using commercial data-scraping agents (Apify YouTube and TikTok Scrapers). Metrics were compared between the AI-generated and overall population datasets using a permutation test [54], a nonparametric test deemed appropriate because of the highly nonnormal distributions of data and the fact that the AI dataset was contained within the overall dataset.

### Qualitative Analysis

A detailed description of the qualitative analysis method is given in the Multimedia Appendix 1. A 2-stage procedure was used. The first stage consisted of an inductive content analysis [55] to categorize features that we deemed educationally problematic. We define “educationally problematic” as violating one or more tenets of effective multimedia instruction according to Mayer’s [51] cognitive theory of multimedia instruction, or principles of quality explanatory video design, including precise and descriptive language, clear learning objectives, and opportunities for engagement and reflection, as outlined by Brame [6], Kulgemeyer [12], and Ring and Brahm [13]. The objects of analysis were decided to be any audiovisual feature (such as graphics, narration, linguistic features, sounds, or combinations of these) that were potentially inaccurate, misleading, distracting, irrelevant, or clearly biased. The videos deemed “likely AI-generated” (plus 6 additional videos found independently; not included in above statistics) were viewed separately, in their entirety, by 2 faculty (EMJ and JDN), one of whom was not involved in the screening steps (the additional videos were found in a YouTube video search for an unrelated project, using queries “enzyme catalysis,” “metabolic pathways,” “what is an enzyme mechanism,” or “lipid bilayer structure”). Each viewer independently compiled a list of such features in all videos and then compared observations. Features tended to relate to either the arrangement and layout of audiovisual and linguistic elements (structural or design features) or the informational content of the videos (content features). An effort was thus made to divide all problematic features between these categories. This was deemed insufficient because certain features involved an inappropriate pairing of structural with content elements. A third category of “content – structure/language” features was thus created for audiovisual features that were “conditionally” problematic based on content, or vice-versa. These categories formed the core of the coding frame. The viewers independently assigned preliminary codes to features in each category, rewatched videos, and modified as appropriate. The viewers then met again to compare codes, re-view videos, and revise until agreement was reached on all codes.

Following the inductive content analysis, a deductive coding stage was performed [56], in which the viewers separately mapped the consensus codes onto the 7 characteristics of careless speech [38]. Reviewers independently assigned codes from the first stage to these 7 characteristics (except “lack of references to source material,” as references are not typically provided in teaching videos), then met and discussed to obtain internal consistency. Some of the codes could not be mapped to the characteristics of careless speech, so 2 new characteristics of slop were proposed, and the reviewers again separately assigned codes to these characteristics, met, and revised until agreement was reached.

After completion of the qualitative analysis, an assessment of each video in the AI dataset as “slop” or “not slop” was made. We considered a video “slop” if it contained at least 2 of our codes of problematic content and exhibited at least 1 of the 7 characteristics of careless speech, except “lack of references to source material.” Agreement by both reviewers was required for a “slop” assignment. All videos in the likely-AI dataset were judged to be slop by these criteria.

### Ethical Considerations

Because this study uses only publicly available data and videos shared with the public are accessible via general search, it is not a research involving human participants, and no ethical review was sought.

## Results

### Summary of Dataset and Prevalence of Slop

**Figure 1.**
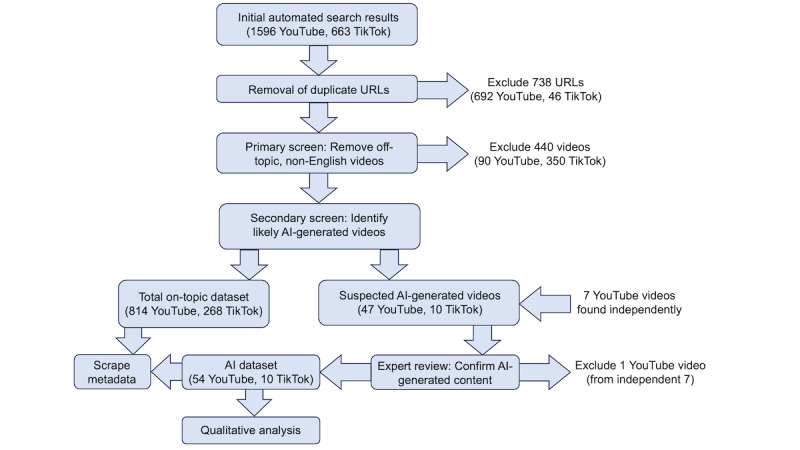
Study design. The final AI dataset for qualitative analysis is a subset of the total on-topic dataset, plus 6 additional YouTube videos found after the initial search (these 6 not included in summary statistics*)*. AI: artificial intelligence.

The study design is summarized in Figure 1. Summary statistics for the 814 YouTube and 268 TikTok videos examined, and a complete numbered listing of all videos, are available in the Multimedia Appendix 2. Regarding RQ2, 47 of 814 YouTube videos (5.8%) were judged to be slop according to our definition. We found that slop on YouTube was concentrated among YouTube Shorts, short-format videos that play in a loop: although only 279 of the YouTube videos examined (34.3%) were Shorts, 37 of 47 videos identified as slop (78.7%) were Shorts, with only 10 standard YouTube videos being slop. This finding is unsurprising, given YouTube’s recent integration of genAI tools with Shorts [57], although most of the videos on the list predate this development. On TikTok, 10 of 268 on-topic videos (3.7%) were judged to be slop; across both platforms, the proportion was 57 of 1082 videos (5.3%) slop. We caution that these numbers likely underestimate the true prevalence of slop, as our method was designed to only identify obvious, low-quality AI-generated videos, and many better-quality videos may have been missed. Furthermore, since the platforms were searched using an automated tool, links to suggested videos, possibly containing more slop, were not retrieved.

**Figure 2.**
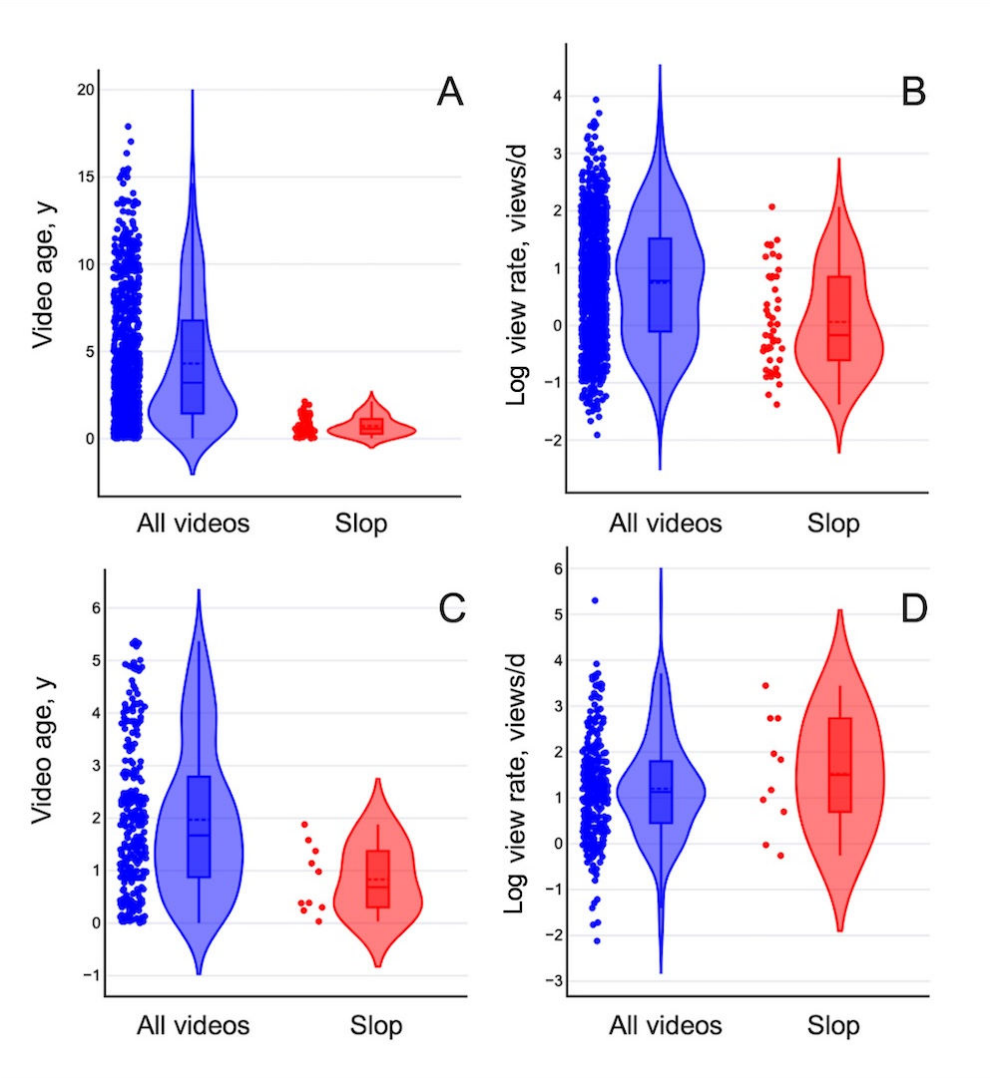
Distributions of the age of videos at time of collection (A and C) and log of view rate in views/day (B and D) for YouTube (A and B) and TikTok (C and D). Dashed and solid lines are mean and median, respectively. Violin plots generated using StatsKingdom Violin Plot Maker.

Regarding RQ3, video metadata revealed the videos varied widely in terms of age (number of days online at time of data collection), duration, number of views (“plays” on TikTok), and number of likes and comments (Tables S1 and S2 in Multimedia Appendix 1). Slop video durations on YouTube were, on average, shorter than the population at large, due to the overrepresentation of YouTube Shorts. On TikTok, the opposite was true; however, the TikTok average is skewed by 1 very long (24 min) video. View and like rates were calculated by dividing the total number of likes, views, and so on for each video by the age of the video in days to obtain average views, likes, and so on per day. This step was essential due to the widely varying ages of the videos, making raw counts of these figures misleading. The distributions of video age and view rate (log scale) are presented graphically in Figure 2; rates of likes and comments are not shown because many videos had no likes or comments and would thus not appear on a log-scale plot.

On both platforms, slop videos tended to have lower rates of engagement (views, likes, shares, and comments) than the population at large, although the difference was not statistically significant (eg, *P*=.87 for TikTok collect rate) according to permutation tests. The difference in engagement was more pronounced on YouTube. YouTube slop videos were engaged with about an order of magnitude less frequently, on average, than the population (Table S1 in Multimedia Appendix 1, last 3 columns), although the extremely broad and asymmetric distributions made the difference insignificant (*P*=.11 for view rate; data not shown). Notably, 21.3% (10/47) of the slop YouTube videos had no likes, and 78.7% (37/47) had no comments at the time of scraping.

Engagement with TikTok videos was generally higher than with YouTube videos (last 4 columns of Table S2 in Multimedia Appendix 1). Rates of views (plays), collects (which we regard as analogous to YouTube likes), and comments are all higher than the corresponding YouTube metrics, which may reflect broader differences in the manner of use of the 2 platforms, or simply a greater number of videos to choose from on the much larger YouTube. TikTok also has a “share” feature, which lacks a direct YouTube analog. All of these metrics were lower in the slop group than in the population (Figure 2D and Table S2 in Multimedia Appendix 1), although the differences were less pronounced than on YouTube (and, again, statistically insignificant; eg, *P*=.38 for view rate; data not shown). All numbers should be taken with caution due to the small sample size (n=10) of slop TikTok videos. Slop thus appears less popular than general materials on TikTok, although proportionately more so than on YouTube. The reason for the difference in relative visibility or popularity of slop between the 2 platforms is not clear from our data. Specifically addressing RQ3, it appears none of the metrics we collected correlates significantly with the presence of slop.

### Qualitative Characteristics of Slop

#### Overview

The qualitative analysis resulted in 16 codes for problematic features. Code categories encompassed features of either the video content, the video structure or format, or features with both structural and content components (ie, structural features not suited to the content). We assigned these categories as groups A, B, and C, respectively, organized hierarchically in Table 2 (the distributions of these codes among our video dataset are given in Multimedia Appendix 2, along with a precise definition of each code, including inclusion and exclusion criteria). Following is a brief description of each code, with examples where appropriate.

**Table 2.** Codes for problematic features of AI-generated videos in our dataset. The “common variants” list is not exhaustive.

Category and codes	Common variants
Content codes	
A1. Factual inaccuracies	Hallucinations or inventions presented as truthful
A2. Omissions of facts or context	Missing details or definitions Missing facts Lack of examples Lack of adherence to best practices of discipline
A3. Overgeneralization or oversimplification	Superficiality Lack of qualifications or contexts for facts Inappropriate conflation of distinct items
A4. Inappropriate or inconsistent level of depth or inattention to audience needs	Uncertain target audience or purpose Missing, unmet, or unclear learning objectives Mixing of beginning and advanced topics
A5. Sloppy analogies	Meaningless analogies (lack of correspondence) Misleading or misemphasized analogies (distracting correspondence) Overextended analogies
Structure and language codes	
B1. Poor graphic or animation quality	Poor clarity, resolution, or size Animation artifacts
B2. Poor audio quality	Inappropriate volume or speed Compression or conversion artifacts
B3. Poor grammar and vocabulary	Nongrammatical speech or text Limited or repetitive vocabulary
B4. Speech or narration irregularities	Inconsistent or unnatural tone, pitch, or emphasis Unnatural pace, cadence, or stress Mispronunciations “Script read aloud” narration
B5. Poor editing or sequencing	Excessive transitions Video and audio transition asynchronously Abrupt beginnings or ends Inappropriate speed
Content – structure and language codes	
C1: Problematic descriptiveness	Overuse of descriptive words and clichés Verbose scripts Indirect, repetitive language Needless or inappropriate emotion Vague, empty descriptions
C2: Mismatching audio-visual elements	On-topic but irrelevant graphic elements Graphics and narration covering different aspects of subject Narrator – narration mismatch Text unrelated to content or speech
C3: Distracting or off-topic material	Music Distracting visual items (eg, watermarks) Needless text overlays
C4: Meaningless graphics	Nonsense graphics or diagrams Nonphysical depictions of physical objects
C5: Text irregularities	Garbled text Illegible text
C6: Disorganization	Illogical sequence of material Poor flow or fluency Lack of linkages between topics or sections

#### A1: Factual Inaccuracies

This code refers to direct errors of fact, that is, hallucinations of the genAI. Factual errors were fairly common in the slop dataset. Some are glaring (eg, Video 1088 claims that biochemistry “allows the sun to rise and set”), but most are subtle and plausible-sounding. For example, Video 546 (nominally about protein structure) discussed “primary,” “secondary,” “tertiary,” and “quaternary proteins,” as if these labels refer to types of protein rather than organizational levels of protein conformation. Video 1083 states that the rate of an enzyme-catalyzed reaction increases, but only up to a limiting value, as the enzyme concentration increases (this is only true if the enzyme concentration exceeds the substrate concentration, which is almost never the case; typically, the rate increases up to a limiting value as the substrate concentration increases, ie, the Michaelis-Menten model). As this latter example illustrates, errors of fact often coincided with misframing of facts (next code).

#### A2: Omissions of Facts or Context

This code reflects content that is narrowly or technically correct, but which does not present needed additional information, that is, misleadingly framed content. This may take the form of missing facts, details, or categorizations, a lack of examples, a lack of nuance or uncertainty, bias, or a lack of adherence to best practices in presenting the subject matter. For example, Video 15 discusses the cytoskeleton keeping cells from collapsing without mentioning that this only applies to eukaryotes, as prokaryotes use the cell wall for this purpose; Video 510 states enzyme active sites fit substrates “perfectly,” which is true only for a small subset of enzymes.

#### A3: Overgeneralization or Oversimplification

Several videos made generalizations about phenomena with important exceptions or simplified topics to a misleading degree. A tendency to generate oversimplified summaries is a known feature of LLMs [58]. Accordingly, videos made oversimplified claims such as quaternary structure being defined as “how multiple protein molecules interact” (Video 691), or treated multiple related topics as a single subject (Video 493, which referred to “urea cycle disorder” as a single disease).

#### A4: Inconsistent Level of Depth or Inattention to Audience Needs

The videos in the AI-generated dataset were extremely diverse in terms of detail, professionalism, and style, and it was often not clear who the intended audience was. Some videos were simply inexplicable, for example, Video 643, a description of the electron transport chain (ETC) atop a clip from the children’s television series “Barney & Friends,” set to a synthesized version of “Yankee Doodle.” Video 875 was apparently intended as a meme. Even the most professional videos, however, often had no apparent audience in mind and covered material to inconsistent levels of detail and depth. Some gave entry-level overviews of a topic, but in a manner that assumed knowledge of more advanced topics (eg, Video 866 ostensibly gives an introduction to the cell cycle, but mentions the functions of maturation-promoting factor and platelet-derived growth factor). Learners would likely find these videos confusing in terms of how much content they should know, or what aspects of the content were most important.

#### A5: Sloppy Analogies

One of the most insidious features of AI-generated videos is the frequency of almost-accurate, yet misleading analogies, which we have termed “sloppy analogies.” In an effective analogy, the items being compared have similar meanings (semantic correspondence) and similar positions or relationships toward other items (structural correspondence), and ideally do not have coincidental, misleading similarities (for detailed discussion, refer to the study by Thagard [59] and references therein). Sloppy analogies violate one of these correspondences (typically the structural correspondence), or make use of distracting, irrelevant similarities between analogs, or extend the analogy to situations where it is not helpful. A learner may thus gain a misrepresentation of the subject, or an improper sense of importance of an irrelevant feature of the subject.

Some sloppy analogies were particularly terrible. For example, Video 679 compares nicotinic acetylcholine receptors to a gateway leading into a beehive, with acetylcholine molecules as “worker bees” that open the gate to allow the “queen bee” (a Na^+^ cation) into the hive (the cell). While the semantic correspondence (the ion channel and the gate) is sound, the structural correspondence is nonexistent: beehives do not have gates, a queen bee does not need to be “let in” to the hive (a queen seldom leaves the hive), and worker bees, unlike acetylcholine, have numerous roles both inside and outside the hive (cell), which they can enter and leave freely. This analogy is baffling, not illuminating.

However, not all sloppy analogies are this obviously bad. Video 1085, for instance, compares metabolic regulation to the coordination of an orchestra by a conductor (“just as a conductor ensures each instrument plays in harmony, enzymes coordinate the complex symphony of biochemical reactions in our bodies”). Both structural and semantic correspondences exist between a conductor and a regulatory enzyme, but the analogy suggests enzymes somehow “choose” which pathways to accelerate or inhibit. It ignores the distributed nature of most metabolic regulation and implies a single, central locus of metabolic control. This analogy captures only one similarity between analogs, ignoring several major dissimilarities, and thus creates a misleading view of how metabolic regulation works in reality.

Other sloppy analogies in the dataset included the ETC being likened to a “relay race” (several videos), protein quaternary structure as a “protein party” (Video 864), and enzymes being akin to a “set of instructions” for making molecules (Video 752).

#### B1: Poor Graphic or Animation Quality

**Figure 3.**
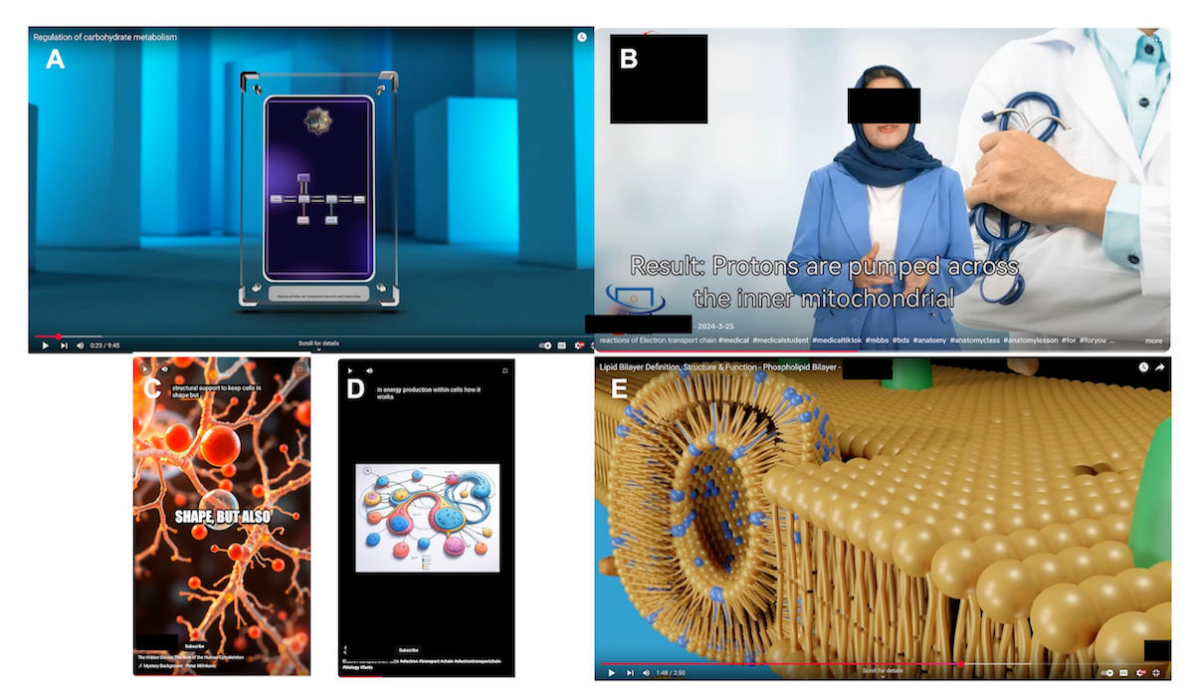
Gallery of video stills illustrating problematic video features. (A) Video 388, diagram too small to be read, unrelated graphic background (codes B1, C2). (B) Video 1045: Monotonous narration, unrelated graphic background, narrator moves and gestures unnaturally (codes B4, C2). (C) Video 559: Distracting text overlay, nonphysical depiction of cytoskeletal fibers (codes C3, C4). (D) Video 712: Meaningless metabolic pathway diagram with garbled text (codes C4, C5). (E) Video 1086: Nonphysical depiction of physical objects (vesicle embedded in bilayer; code C4). Names and logos of content creators have been redacted.

Most videos in the dataset made use of cartoon graphics or animations, or joined still images with transitions. In making the videos, insufficient attention to detail or editing led to poor-quality graphics, such as low-resolution, pixelated, or blurry images, pictures too small to be clearly seen, and jerky or flickering movement (Figure 3).

#### B2: Poor Audio Quality

Similarly, inattention to editing caused many videos to have audio that was too fast or slow, varied wildly in volume, or contained sudden cuts. This code applies to general audio; spoken language is accounted for by codes B3 and B4.

#### B3: Poor Grammar and Vocabulary

Several videos contain grammatical errors in either spoken words or on-screen text (eg, “Do you know what is cytoskeleton?” [Video 222]) or use inappropriate vocabulary. Most of these errors are minor and unlikely to affect understanding of the subject, but are distracting to native English speakers and may be confusing to learners whose native language is not English.

#### B4: Speech or Narration Irregularities

Many videos with spoken narration exhibit the flaws commonly seen in text-to-voice translation: Unnatural tone or stress, mispronunciations of certain words, awkward pace or cadence, emotionless (or overly emotional) tone, and narration that sounds like text being read aloud. Abbreviations are often read out like words, for example, K_A_, association constant, was pronounced “ka” in Video 864; in Video 712, ETC (electron transport chain) was read as “et cetera.” These flaws are generally not sufficient to affect understanding of the material, but they are distracting and require extraneous processing to ignore [6].

#### B5: Poor Editing or Sequencing

Videos in the list often exhibited excessive or poorly executed transitions between images or sections, started or stopped abruptly (often mid-sentence), shifted rapidly between different topics, or featured visual and auditory elements that did not transition together, leading to an audio-visual mismatch (also refer to code C2). In most cases, this lack of attention to fluent editing created only a distraction; poor sequencing impacting understanding of the content is captured by code C6.

#### C1: Problematic Descriptiveness

Several studies have found LLM-generated writing tends to overuse adjectives [60] or create prose with an effusive, grandiose style [61,62]. We observed this tendency in nearly all of the videos in our AI-generated dataset, most of which were apparently based on LLM-written scripts. Words frequently overused by AI (“amazing,” “crucial,” and “delve”) were superabundant. In addition to descriptive words, the videos in our list frequently overused certain cliches [63]: “deep dive,” “break it down,” “unsung hero” (Video 1085 used this term four times in nine minutes), and constructions like “from … to …” (or “whether it’s … or …”) all appeared far more frequently than would be expected in ordinary narration. More generally, the scripts of our videos tended toward repetitive and indirect language, often incorporating needless emotion or forced casualness (“pretty cool, huh?”). In extreme cases (Video 1088), the scripts gave elaborate, emphatic declarations of a topic’s importance without ever incorporating actual facts. Several videos also made analogies for simple concepts not needing an analogy, such as the cytoplasm filling the inside of a cell “like the water in a water balloon” (Video 638; also refer to code A5).

#### C2: Mismatch of Audio and Visual Elements

In multimedia educational materials, visual elements should be paired with relevant auditory elements so that inputs to the 2 cognitive channels can reinforce one another; off-topic and unnecessary visuals should be minimized [6]. This principle was frequently violated in the AI-generated videos in our dataset. Off-topic graphic backdrops were present in many videos (Figure 3A–B), and in others, the graphics and narration described different aspects of the subject, or text not matching or reinforcing the narration was displayed. Video 1087, for example, showed a model of DNA while discussing proteins. Some videos (eg, Videos 940 and 1087) displayed animated or avatar narrators whose hand gestures did not match the points of emphasis in the script (Figure 3B), a common artifact of photo-animation software like HeyGen. These distracting visual elements require cognitive processing to ignore, diluting the educational effectiveness of the videos. They may also, without careful structuring, suggest misleading connections between audio and visual content, resulting in a misconception of the topic being presented [64].

#### C3: Distracting or Off-Topic Material

Beyond mismatched visuals and sound, many videos displayed miscellaneous off-topic and distracting features, such as music, watermarks, animations, or unnecessary text overlays (which often obscured relevant imagery). Some videos included unrelated or loosely related stock footage (eg, Video 541, about fatty acids, showed supplement pills on a tray). These so-called “seductive details” contribute to cognitive load without imparting real information [65].

#### C4: Meaningless Graphics

Some of the visual elements in the videos were nonphysical representations of real objects, or completely meaningless diagrams (Figure 3C–E). Many of these graphics could be scientifically misleading, such as an inaccurate rendering of a protein structure (Video 691) or a phospholipid vesicle embedded in a bilayer membrane (Video 1086, Figure 3E).

#### C5: Text Irregularities

AI image generators struggle to create realistic text, and accordingly, several of the videos featured nonsense words resembling real words, such as “eectron.” Properly rendered text was also often illegible, either due to poor resolution, inadequate size, or cropping.

#### C6: Disorganization

Videos in the dataset frequently suffered from general disorganization. Topics were presented in a nonintuitive sequence, concepts did not flow naturally from one to the next, and linkages between subjects were often not explained or insinuated. Disorganization occurred at the level of structure and editing (eg, a rapid series of images being flashed in the background while a single topic was explained, as in Video 638) or content (eg, Video 1083 described the effects of enzyme inhibitors on an enzyme’s kinetic parameters before discussing enzyme kinetics). Disorganization contributes to cognitive load by requiring the learner to “hold” relevant information in working memory while waiting for complementary information [65].

Many of the 16 codes of problematic slop content could be directly matched with the seven characteristics of careless speech: (1) factual inaccuracies or inventions (hallucinations or obsolete ideas); (2) nonrepresentativeness of sources (bias; statements not proportionally representing the totality of views); (3) incompleteness (statements that are narrowly correct but omit needed context); (4) lacking signifiers of uncertainty (unwarranted confidence or failure to account for variability in responses); (5) lacking references to source material (failure to cite relevant sources, where appropriate); (6) references not based on referred text (hallucinated or off-topic references); and (7) inaccurate summaries of referenced text (incorrect or incomplete summary of a real reference) [38]. These mappings of codes are presented in Table 3.

**Table 3.** Alignment of qualitative codes from Table 2 with characteristics of careless speech. Two additional code groupings, which we designate “communicative nonfluency” and “message – delivery incoherence,” were identified in addition to the 7 features of careless speech published previously.

Careless speech characteristic	Codes from Table 2
Factual inaccuracies	A1, A2, C4, C5
Nonrepresentativeness of sources	A2, A3
Incompleteness	A2, A3, A4, A5
Lacking signifiers of uncertainty	A3, A5
Lacking references to sources	A2
References not based on referred text	A1, A2
Inaccurate summaries of referred text	A1, A2, A3, C1, C4
Additional characteristics of slop	
Communicative nonfluency	B1, B2, B3, B4, B5, C6
Message – delivery incoherence	A4, C2, C3, C6

Because the features of careless speech describe content, they align most closely with groups A and C of our codes for characteristics of slop. For instance, A1 is virtually identical to “factual inaccuracies or inventions.” We note that the careless speech codes involving references are less relevant to the case of educational videos or lessons, which often do not cite references (references are presumed to be the latest discipline-standard textbooks or review articles). Only 2 of the videos in our slop dataset (681 and 697) cited a reference.

The group B codes, and codes C2, C3, and C6, did not specifically align with any of the characteristics of careless speech, as these codes are primarily concerned with the form and structure of the speech. We consider these codes to embody 2 additional common characteristics of slop (if not of careless speech more generally): “communicative nonfluency” (C6 and all group B) and “incoherence of message and delivery” (A4, C2, C3, and C6). Communicative nonfluency often makes low-end genAI materials recognizable (telltale linguistic features, robotic narration, unnatural animations, and so forth), while the incoherence of message and delivery (features such as excessive or unnecessary transitions, mismatching visual and auditory output, confusing or inappropriate diagrams, and illogical sequencing) limits its usefulness as a teaching tool, even when factually accurate. Since many videos incorporated content or styles that were incompatible with a particular audience or set of learning goals, we included code A4 in this group as well.

## Discussion

### Prevalence and Reach of Slop

Our results show that slop accounts for a small but nonnegligible portion of medical biochemistry and cell biology videos, seems to be comparably popular to nonslop videos, and cannot be reliably distinguished from nonslop on the basis of that quantitative features. These findings accord with previous studies of educational YouTube videos, which found quantitative metrics do not correlate with video quality [11,66]. Some studies have suggested that the number or text of comments may correlate with quality [11], but we did not observe any strong correlation of comment rates with slop (Tables S1 and S2 in Multimedia Appendix 1). The text content of comments was not examined in this study. We may thus conclude that slop cannot easily be identified without viewing the video. We also emphasize that our method is only able to detect obvious and low-quality genAI output, so the true prevalence of slop is certainly higher than our numbers suggest, and as the apparent quality of genAI content improves, even viewing a video may soon be insufficient to identify it as slop.

In the course of this research, we observed that many of the slop videos were posted by channels that consisted mostly or entirely of slop material, suggesting that characteristics of the channel or creator, and not the individual video, may provide evidence that a video is slop or otherwise questionable. Studies of channel characteristics, rather than video characteristics, should thus be a productive line of future slop research.

### Problematic Features of Slop

At present, research on the educational effects of genAI video is scant. At least 3 recent small-scale studies have examined the effectiveness of AI-constructed video on learning, and all found little difference in learning outcomes between genAI and traditional materials [67-69]. Critically, however, all of these studies used extensively edited and fact-checked videos, designed by disciplinary experts. In other words, the videos in these studies were not slop. A full understanding of the hazards of slop must be drawn not from well-designed genAI materials but from slop typical of online video platforms.

When addressing RQ1, we identified 16 problematic features of slop videos that may impact educational value (Table 2). These features encompass both subject matter and structure-based aspects, and thus imperfectly align with the features of careless speech (Table 3), which is a subject matter–based construct. We feel it is relevant to include structure-based features in consideration of slop, since proper formatting and editing of multimedia educational materials contribute to educational effectiveness [6,12,65,66]. Thus, some consideration of video format and structure is appropriate in assessing the impact of slop.

While all 16 features can dilute the educational effectiveness of videos, we think 2 deserve additional discussion. The first of these is sloppy analogies (code A5). The educational perils of imperfect analogies and metaphors have been described for disciplines including the biological sciences [70,71], chemistry [72,73], and physics [74,75]; genAI does not add any new hazards. Rather, genAI removes the effort barrier to creating a weak analogy, and along with it, the mental check of whether the analogy makes sense (unless further prompting or editing, ie, care, is performed by the content creator). What is particularly damaging about sloppy analogies is the illusion of understanding [76] generated by an intuitive but inaccurate or unnuanced analogy. Previous studies have shown that analogies can have negative effects on metacomprehension if not reinforced by experiential inputs, such as experimentation [77]. Video, being an inherently passive medium, is thus especially inclined to mislead by analogies presented without real-world reinforcement, and experimental data confirm that video instruction is prone to an illusion of understanding effect when misconceptions are present [76,78]. While this is equally true of all videos (not just slop), the ease with which genAI conjures plausible-sounding analogies makes slop videos especially likely to contain poor analogies and metaphors, as seen in our dataset. Incidentally, at least 1 popular book on genAI in teaching [79] specifically recommends asking an LLM to create analogies for unfamiliar topics. Based on our results, we strongly feel unsupervised beginning learners should not follow this suggestion (to be fair, this book emphasizes the importance of careful prompting when asking an LLM for an analogy, but since beginners are typically not capable of evaluating the analogy, there would be no way for a beginner to know if a prompt was effective). However, experienced instructors might find this suggestion useful as part of a carefully curated activity, for example, asking students to identify problems with the analogy.

The second educational hazard worth further discussion is problematic descriptiveness, code C1. Numerous studies have commented on the tendency of LLMs to overdescribe [60,62] as in our slop videos. For example, the passage “a toolbox of folds and domains that evolution has mixed and matched over billions of years to create the incredible diversity of proteins we see today” (Video 864) has to be read or heard several times to extract the main point: that proteins contain modular folds and domains that perform discrete functions. Apart from being distracting (and annoying), this overly descriptive style creates a problem of misplaced emphasis in many of the videos. Unable to know which terms or ideas are really important and which are incidental, the LLM attaches descriptive words or phrases to everything it can. A human teacher, however, would preferentially reserve description for the most salient words and topics, thus cueing the learner toward the important material. Goodwin [80] described this practice as “highlighting,” and identified it as one component of the so-called professional vision that frames expertise in any discipline. An LLM lacks the professional vision of an educator (or any other profession) and thus cannot model a professional’s practice of sense-making, except insofar as a human professional shapes the genAI output. Instead, all aspects are treated as potentially equal by the LLM, resulting in an unfocused, directionless treatment of the subject.

Other features of slop identified in our qualitative analysis generally align with the 7 features of careless speech [38] or violate good practices of multimedia teaching [6,51]. We identify 2 clusters of features that do not neatly map onto the careless speech framework, “communicative nonfluency” and “message – delivery incoherence” (Table 3). Communicative nonfluency may be loosely defined as the property of requiring undue cognitive effort to understand; it aligns closely with so-called perceptual fluency, or the ease of making sense of inputs based on sensory features [81]. Perceptual fluency is strongly associated with metacognition, specifically judgment-of-learning [82] and perceived accuracy or truth [83,84]. Accordingly, students have perceived fluent delivery (in video or live lecture) as more instructive even though fluency did not affect learning gains [85,86]. These findings suggest students prefer fluent over nonfluent learning materials, and thus would be less likely to perceive slop videos as reliable, even if the slop video lacked any factual errors. However, as the realism and fluency of genAI output increase, this effect would be expected to diminish as technology improves, and thus communicative nonfluency may not be a characteristic marker of slop in the future.

Message – delivery incoherence refers to a mismatch between the concept being communicated (the message) and the object or language ostensibly used to communicate it (the delivery). In our video dataset, this most frequently took the form of mismatching audio and visual elements (code C2) or superfluous content (code C3). Either of these will increase the amount of cognitive processing needed to encode the underlying message, and thus hinder learning [65]. For instance, extraneous visual content (such as watermarks and text overlays duplicating the narration) conveys no relevant information and competes for working memory with the educational content [6]. Likewise, mismatched content between channels (such as a description of cell cycle regulation over a schematic of ligand-receptor binding; Video 1051) requires processing to identify which channel contains the relevant information, so-called extraneous overload [65], or may create a misimpression that the two channels are, in fact, related. It is thus considered best practice in video instruction to remove superfluous material (“weeding”) and to ensure information in audio and visual channels complement each other [6,65]. Message – delivery incoherence also sometimes took the form of presentations that were inappropriate for the apparent learning goal of the video, or were so muddled that the learning goal was indiscernible (codes A4 and C6). Relevance to learners and links to previous knowledge are considered important elements of effective instructional video design [13], and were conspicuously weak in most of the slop videos. These deficiencies could impact engagement with the videos [6], even if factual accuracy were not a concern.

Of course, none of our problematic features of slop is entirely unique to AI-generated material, and most are not unique to video. Classroom lectures may use flat, repetitive language or incorporate unnecessary content; human teachers may make bad analogies or fail to highlight key points. Living instructors, however, generally bear some risk of consequence for ineffective teaching (care in the second sense of the term), such as poor evaluations, career stagnation, or a personal sense of failure. GenAI is totally unencumbered by such consequences, and the human who uses genAI to make educational slop for free public platforms (particularly when posting anonymously) is, to some extent, insulated from these consequences—although not from gain in the form of monetization or self-satisfaction. Additionally, living instructors often have the chance to immediately correct student misconceptions that may result from a bad analogy or other errors through just-in-time teaching techniques; for asynchronous video, this is not a possibility, requiring videos to be high-quality from the beginning. Slop is likely to remain a significant problem on video-sharing platforms as long as there is an asymmetry between the risks and rewards of sharing it. Students lacking the expertise to evaluate content on unfamiliar subjects will be especially vulnerable.

### Implications for Content Creators and Learners

Fortunately, our inventory of slop characteristics provides a ready checklist of pitfalls that caring creators of genAI material can work to avoid. The central pillar of making good genAI material is maintaining a human-in-the-loop [44] at every point in the creation and dissemination process. Prompting (and reprompting or iterative prompting) needs to be done in a planned and deliberate fashion; tools such as prompt-design frameworks [87] are helpful at this stage. The output must then be evaluated not only for accuracy and appropriate context, but also for alignment with intended learning goals and audience characteristics, which should be explicitly stated. GenAI descriptions, explanations, and metaphors should never be accepted at face value, but rather examined closely (and amended, if necessary) to avoid misleading, oversimplified, or misemphasized statements. If possible, the output should align with the viewer’s experience of reality and use meaningful mental models (eg, the human experience, expertise, accuracy, trust, or HEAT heuristic [44]) and be coherent with respect to subject matter and presentation. The output should give a balanced overview of the field without unduly favoring a particular viewpoint [41]. Attention should be paid to the fluency of the output (video quality and realism, natural-sounding speech and audio, and appropriate transitions). Once posted to an online forum, material should be monitored for signs of misinterpretation (eg, comments reflecting confusion or dislike) and edited or removed as necessary. Finally, clear disclosure of the use of genAI will help establish trust with the audience. While this will not impact the quality or effectiveness of the material, these efforts toward building trust may be seen as a sign of care. Slop-free genAI material requires more than just good prompting; human involvement throughout the lifecycle of the material is essential to make sure good AI creations continue to fulfill their intended functions. We are currently in the process of developing lifecycle guidelines for genAI educational materials.

For learners, the presence of slop on video-sharing sites presents a challenge. The videos encountered in the course of this study are at the lowest end of quality and usefulness; future slop may be far more realistic and seemingly helpful than today’s slop, but as long as genAI works in an associative fashion, it will always be careless speech (and thus unreliable). Learners should thus focus not on detecting and excluding suspected genAI material, but on cross-checking claims made in online videos with other sources, verifying the credentials of the creators, and discussing points of concern with subject matter experts. Sadly, vetting and comparing sources of information is a skill that takes time and effort to develop. Furthermore, genAI is often presented to learners as a shortcut to learning (eg, as a way to quickly digest and summarize dense information), or as a source of information [39] while its ability to introduce misconceptions and misframings of reality [28,38] is far less recognized. We therefore recommend that learners approach known genAI output with great caution, always validate sources, and consult with experts when possible, and generally exercise judgment when using video-sharing platforms for educational purposes.

### Limitations

Our definition of slop is intended only for educational materials and may not be appropriate in other contexts, such as art, advertising, or political speech. Since we restricted our attention to videos, not all characteristics of slop may be relevant to other output types (such as still images or text). Our numerical data should be considered semiquantitative at best, since we believe our methods underestimate the prevalence of slop for reasons given above. Our estimates of slop’s prevalence for RQ2 may not apply to other major platforms where slop is endemic (eg, Facebook [Meta] or Instagram [Meta]) due to the different user bases and content moderation practices on these platforms. We should also emphasize that our aim in RQ1 was only to identify problematic features of genAI material—any potential educational benefits of AI-generated learning materials were not considered for the purposes of this study.

A well-known problem with genAI output is bias, which in this study was encompassed by code A2, incompleteness. We did not examine bias in our dataset in any further detail (eg, biased overviews of a discipline and bias in examples), but this aspect of slop clearly needs deeper attention in future work. We also note, as mentioned previously, that our own dataset is biased toward obviously bad genAI content, due to our screening method, so some of our findings may not be fully generalizable to higher-quality slop.

Our methodology made no effort to identify the purposes for which the slop videos were made. It is likely that most of the AI videos in our dataset were made not to educate, but to accumulate views and other markers of engagement. While irrelevant to the educational impacts of slop, further study of the motivating factors behind slop’s rapid proliferation on the internet may help to curb its influence.

Perhaps most significantly, this study does not address the enormous ethical concerns raised by slop, such as unmitigated biases, the unpaid and uncredited use of intellectual property for training, and the environmental impacts of needless AI use [41,50]. Slop disregards ethical considerations, as care forms a basis for ethics [46], and a definition of slop that is based in an ethics framework (rather than on features of its content, as in this study) would be needed for a proper accounting of the ethical dimension of slop. Regardless, ethical matters feature prominently in UNESCO’s (United Nations Educational, Scientific and Cultural Organization) recent statement of guidelines for responsible AI use in education [88], and while ethics is only one of many risks of improper educational use of genAI [50], it deserves attention in future research on slop.

### Conclusions

We have suggested a definition of slop that, to our knowledge, is the first in the scholarly literature. We define slop as AI-generated material that is produced with little or no care toward its educational usefulness or quality, situated in the broader conceptualization of careless speech [38]. Using this definition, we find that slop composes a small percentage of preclinical biochemistry and cell biology videos on YouTube and TikTok, but these videos could be found without considerable effort and present specific educational risks to learners. Among these risks are misleading content (eg, inaccuracies and improper comparisons), nonfluency endangering effective cognitive processing, and incoherent presentation (eg, problematic descriptiveness and disorganization). Our findings can hopefully inform better practices among responsible creators of genAI material for education. We also hope our findings will be informative to other disciplines, such as journalism, in which vetting of information sources is critical and under threat by genAI materials.

Some commentators [16,24] have suggested that slop is a temporary problem that will be solved by technological means, much as spam email has been curtailed by email filtering. Even if this proves to be true, existing slop videos will persist on the internet for some time, potentially training future genAI applications, and thus propagating subtle misconceptions or misemphases throughout future, higher-quality genAI output. As genAI becomes more central to education and training, these propagated errors may be used to train human beings, leading to a generational erosion of understanding and expertise [38]. Slop is therefore a problem that needs to be recognized and fought right now, and we hope this study provides a useful starting point for this fight.

## Supplementary material

10.2196/80084 Multimedia Appendix 1 Detailed methods and descriptions of qualitative codes.

10.2196/80084 Multimedia Appendix 2 Complete listing of videos in datasets with URLs and content codes.

## Abbreviations

AI: artificial intelligence
API: application programming interface
ETC: electron transport chain
genAI: generative artificial intelligence
LLM: large language model
RQ: research question
