# Supplementary material for: AI-Generated “Slop” in Online Biomedical Science Educational Videos: Mixed Methods Study of Prevalence, Characteristics, and Hazards to Learners and Teachers
Source: JMIR Med Educ. 2025 Nov 20;11:e80084. doi: 10.2196/80084 (PMC12634010; doi:10.2196/80084)
Supplement: Multimedia Appendix 1 [file mededu-v11-e80084-s001.docx]

**Contents of this file:**

1. Appendix S1. Detailed methods: Collection and screening of videos, screening for signs of AI use, qualitative content analysis
2. Appendix S2. Descriptions of qualitative codes with inclusion and exclusion critera
3. Table S1: Descriptive statistics for YouTube videos
4. Table S2: Descriptive statistics for TikTok videos

**See also:** Jones et al File F1 video list.xlsx (complete listing of all videos in dataset with URLs and assigned codes)

**Appendix S1. Detailed methods**

***Collection and screening of videos:*** YouTube was searched using the SerpAPI YouTube Search Engine (<https://serpapi.com/youtube-search-api>), set to the English language and with caching disabled, in a private window of the Firefox 128.7 browser, with history and cookies deleted prior to the search.  Because YouTube uses continuous pagination, the pagination (“sp”) parameter was set to “next_page_token” so that multiple pages of results could be collected.  Ten different search queries pertaining to different biochemistry topics were entered (listed in Table S1), and for each, the top three pages of results (consisting of 20-30 links plus a few “hidden” links) were output in JSON format.  From the JSON files, bare URLs were extracted to an Excel sheet and duplicate entries were removed.  The resulting list of 908 unique URLs was then subject to a preliminary screen, in which a few seconds of each video was watched by one researcher to verify the material was in English and on-topic.  After removal of irrelevant, non-English, and unavailable videos, 814 remaining videos were subject to a secondary screen to search for likely AI-generated material, using linguistic and audiovisual “tells” commonly seen in such media (see below). For the secondary screen, each video was watched by one researcher for a minimum of 30 seconds (or the entire video, if under 30 seconds), and videos suggestive of AI output were compiled in a final list of 47 likely AI-generated videos for final analysis.  Metadata for all 814 videos passing the preliminary screen (including date posted, number of likes, views, subscribers, and comments) were scraped using the Apify YouTube Scraper agent (<https://apify.com/streamers/youtube-scraper>) and stored in Excel format.

         TikTok was searched using the Apify TikTok Search API agent (<https://apify.com/epctex/tiktok-search-scraper>), using the same search queries as for YouTube and with a limit of 60 videos per query (though in some cases more than 60 hits resulted, for unknown reasons).  A residential proxy server was used.  From the JSON output, URLs were extracted and screened as described for the YouTube videos, above.  After removal of duplicates, 617 unique video URLs were obtained, of which only 268 were on-topic and in English.  After the secondary screen, 10 videos were flagged as likely AI-generated and were added to the list of YouTube videos for detailed review.  Metadata for the 268 on-topic videos were scraped using the Apify TikTok Scraper (<https://apify.com/clockworks/tiktok-scraper>) on a residential proxy server.  In total, from 1082 on-topic videos screened (814 YouTube and 268 TikTok), 57 were flagged as likely AI-generated, 47 YouTube and 10 TikTok.  For the metadata, population means and distributions were compared using a permutation test [1], a non-parametric comparison appropriate in this case because of non-equal variances and highly non-normal distributions of the datasets, and because the AI dataset was contained within the population dataset.

         The final list of 57 flagged videos, plus seven additional YouTube videos found independently, was distributed among three biochemistry professors who watched the videos separately and scored each video on two measures: Whether or not, based on the “tells” described below, the video was likely AI-generated (0 = not AI-generated; 1 = partially AI-generated; 2 = mostly or entirely AI-generated); and whether or not the video contained any factual errors (Y or N).  If a Y score was applied, a description of the error(s) was provided.  The results were compiled by the lead author, and any video with an average score of 1.0 or above was deemed “probably AI-generated.”  One video was excluded at this point for having an average score below 1; the remaining videos were subject to qualitative analysis.

***Screening for signs of AI generation:*** Videos were accessed from within the Excel sheet of deduplicated URLs. Each video was screened for relevance and for signs of AI use, and was flagged if any of the following features was noted:

- Nonphysical graphics: Impossible objects, incorrect renderings of real objects (e.g. anatomical irregularities, incorrect movements or transitions [2]);
- Speech irregularities: Unnatural or “robotic” style of speech with improper rhythm or emphasis, mispronunciations of words, mismatch of spoken words with visual content [2, 3];
- Linguistic “tells”: Overuse of particular words (e.g. *amazing, exciting, pivotal, delve*) or idioms (*deep dive, game-changer, whether ___ or ___*), vague and indirect style [2-4] (see links below for full listing);
- Direct disclosure of AI use, e.g., a watermark or a note in the description.

AI detection tools were not used due to their tendency to produce inconsistent results [5].  More complete listings of common linguistic “tells” are available from Pangram Labs (<https://www.pangram.com/blog/how-to-detect-ai-writing>) and Science Editing Experts (<https://www.scienceeditingexperts.com/blog/ai-red-flags-an-extensive-list-of-words-and-phrases-that-scream-this-was-written-by-chatgpt>; links accessed 8 May 2025).

***Qualitative content analysis:*** The suspected AI-generated video list was subject to a two-stage qualitative analysis to identify and categorize features that are likely to be problematic for educational purposes. By “problematic,” we mean in violation of best practices in at least one of two frameworks for effective multimedia instruction: 1) proper arrangement of materials and integration of audio and visual elements in order to streamline delivery and minimize extraneous cognitive load, according to Mayer’s theory of multimedia teaching [6]; or 2) appropriate selection of examples, descriptive language, clear learning objectives, illustrative visuals, and opportunities for active engagement, as described by Brame, Kulgemeyer, and others [7-9].

The first stage of the qualitative analysis was an inductive content analysis [10]. Two researchers independently agreed on video elements to be noted as potentially problematic: Individual visual elements, descriptive passages, sounds, and linguistic features (such as pronunciation, word choice, or style) that could potentially be inaccurate, misleading, distracting, irrelevant, or obviously biased. The reviewers then separately viewed all videos in their entirety and compiled a list of such features for each. It was found that some features related mainly to the content of the videos (statements of fact, manner of summarizing or grouping information, etc.) while others related to the design and arrangement of elements (layout of diagrams, use of music or text, etc.) and thus were structure-based. An effort was thus made to divide individual features into structure and content categories. It soon became apparent that not all features could be easily assigned to a content-based or structure-based grouping, as some incorporated both structural and content features, e.g., arrangement of audiovisual items in a manner that was not inherently problematic but was inappropriate for the content. A third grouping of so-called content-structure features (that is, structural features that *conditionally* depend on certain content features, or vice-versa) was therefore created. These three categories formed the core of the coding frame. The reviewers separately assigned preliminary codes to each video feature according to the principle(s) of good multimedia instruction that were violated, re-watched the videos, and modified codes as appropriate. The reviewers then met again and discrepancies in coding were discussed (with re-viewing where needed) in order to reach consensus on codes without having to rely on quantitative metrics. The end result was 16 codes for problematic features assigned among the three categories (Table 2 in the main text). The assignment of codes to each individual video can be found in the associated Supplemental File.

The second stage of the qualitative analysis involved a deductive content analysis [11] in order to map the codes from the first stage to the pre-selected theoretical construct of careless speech. The careless speech theory describes 7 aspects of genAI content that contradict or obscure objective truth [12]. The two reviewers separately assigned codes to any of the seven features of careless speech (except “lack of references to source material,” which was deemed irrelevant for instructional video), met, discussed, and revised assignments to obtain internal consistency. Because careless speech is a content-based construct, the structure-based codes and some structure-content codes could not be matched to any of the seven features, so two new “emergent” categories that are characteristic of slop, but not necessarily of careless speech in general, were defined according to the most convenient groupings of unassigned codes. These groupings (communicative non-fluency and message-delivery incoherence; Table 3 of the main text), together with the characteristics of careless speech more broadly, constitute what we believe to be 9 properties that are hallmarks of slop, as these features demonstrate lack of care in addition to the truth-independence characteristic of careless speech.

**References**

1. Pitman, E. J. G. Significance Tests Which May be Applied to Samples From any Populations. In *to the Journal of the Royal Statistical Society* (Vol. 4, Issue 1); 1937. <https://www.jstor.org/stable/2984124> [Accessed June 23, 2025]
2. Hassan, N.A. How to identify AI-generated content: A step-by-step guide. *Cybernews.* 2025. <https://cybernews.com/editorial/how-to-identify-ai-generated-content-guide/> [Accessed May 30, 2025]
3. Anonymous. Recognize artificial intelligence (AI): 9 ways to spot AI content online. Government of Canada *GetCyberSafe* Resources. <https://www.getcybersafe.gc.ca/en/resources/recognize-artificial-intelligence-ai-9-ways-spot-ai-content-online> [Accessed May 30, 2025]
4. Tiffany, K. Welcome to the Golden Age of Cliches. *The Atlantic*. February 21, 2023. <https://www.theatlantic.com/technology/archive/2023/02/ai-chatbots-cliche-writing/673143/> [Accessed June 23, 2025]
5. Dugan, L., Hwang, A., Trhlik, F., Ludan, J. M., Zhu, A., Xu, H., Ippolito, D., Callison-Burch, C. *RAID: A Shared Benchmark for Robust Evaluation of Machine-Generated Text Detectors*. Arxiv. Preprint posted online June 10, 2024. Doi:[10.48550/arXiv.2405.07940](https://doi.org/10.48550/arXiv.2405.07940)
6. Mayer RE. Cognitive Theory of Multimedia Learning. In: Mayer RE, editor. The Cambridge Handbook of Multimedia Learning. 2nd ed. Cambridge, MA: Cambridge University Press; 2014:43-71. doi: 10.1017/CBO9781139547369.005.
7. Brame CJ. Effective educational videos: Principles and guidelines for maximizing student learning from video content. CBE Life Sci Educ. 2016 Dec 1; 15(es6):1-6. doi: 10.1187/cbe.16-03-0125.
8. Kulgemeyer C. A Framework of Effective Science Explanation Videos Informed by Criteria for Instructional Explanations. Res Sci Educ. 2018; 50(6):2441-2462. doi: 10.1007/s11165-018-9787-7.
9. Ring M, Brahm T. A Rating Framework for the Quality of Video Explanations. Technol Knowl Learn. 2022; 29(4):2117-2151. doi: 10.1007/s10758-022-09635-5.
10. Vears DF, Gillam L. Inductive content analysis: A guide for beginning qualitative researchers. Focus Health Prof Educ. 2022; 23(1):111-127. doi: 10.11157/fohpe.v23i1.544.
11. Hamad EO, Savundranayagam MY, Holmes JD, Kinsella EA, Johnson AM. Toward a Mixed-Methods Research Approach to Content Analysis in the Digital Age: The Combined Content-Analysis Model and its Applications to Health Care Twitter Feeds. J Med Internet Res. 2016;18(3):e60. doi: 10.2196/jmir.5391.
12. Wachter S, Mittelstadt B, Russell C. Do large language models have a legal duty to tell the truth? Roy Soc Open Sci. 2024; 11(8):240197. doi: 10.1098/rsos.240197.

**Appendix S2. Descriptions of qualitative codes with inclusion and exclusion criteria**

Below are listed definitions of each of the 16 codes identified in the content analysis, with inclusion and exclusion criteria. In the case of exclusion criteria, the proper code for each item is given.

**Group A: Content codes**

**A1. Factual inaccuracies:** False statements or depictions stated or shown as truthful.

***Includes:*** Hallucinated/invented facts or references, mathematical errors, incorrect names of concepts or items.

***Excludes:*** Omissions of necessary facts (A2), irrelevant content (C3), meaningless or nonphysical graphics (C4), garbled text (C5).

**A2. Omissions of facts or context:** Failure to include facts or supporting material needed to establish truth, limitations, or usefulness of content.

***Includes:*** Missing details or definitions of objects, missing examples, failure to cover essential aspects of the topic, failure to present in a manner accordant with best practices in the discipline.

***Excludes:*** Inaccurate or incomplete summaries of subject (A3), lack of nuance (A3), insufficient or inappropriate depth of coverage of subject (A4), lack or poor quality of visual elements (B1), lack of narration (B4), descriptions that fail to capture key characteristics of subject (C1).

**A3. Overgeneralization or oversimplification:** Summarization of subject in a manner that incorrectly extends to unrelated areas, or that removes or distorts categorizations, nuance, limits, or important exceptions.

***Includes:*** Superficial coverage of subject, failure to note limitations or exceptions, failure to retain important categorizations, inappropriate use of absolutes (“always,” “every,” etc.)

***Excludes****:* Inappropriate depth for intended audience (A4), inadequate context (A2), inappropriate use of analogy or metaphor (A5), descriptions that fail to capture key features of subject (C1), failure to link concepts or present subject coherently (C6).

**A4. Inappropriate or inconsistent level of depth/inattention to audience needs:** Presentation is not appropriate for intended most likely audience in terms of depth of coverage, consistency in level of presentation, or professionalism; failure to state or imply learning objectives or provide opportunities for learner engagement, reflection, or thought about subject.

***Includes:*** Material covered at too basic or too advanced a level for audience; inconsistent level of depth throughout video; unclear or unmet learning objectives; intended scope of coverage of subject is not clear; unprofessional presentation of subject.

***Excludes:*** Oversimplification of complex subject (A3), use of informal language (B3), inappropriate emotion (B4), unclear or indistinct descriptive style (C1), disorganized structure or linkage of topics (C6)

**A5. Sloppy analogies:** Analogies or metaphors in which objects being compared do not have similar meanings or functions, or do not function similarly with respect to other objects, or have significant dissimilarities or irrelevant similarities that could lead to confusion.

***Includes:*** Inappropriate comparisons of concepts to physical objects or scenarios; comparisons that ignore significant dissimilarities; insufficiently explained analogies.

***Excludes:*** Oversimplifications (A2), inappropriate use of elaborate or metaphorical descriptive language (C1).

**Group B: Structure/language codes**

**B1. Poor graphic/animation quality:** Visual elements are undiscernable or are animated in a manner that is distracting or nonphysical.

***Includes:*** Graphics too small or stretched to be discerned clearly; animated features (including avatar narrators) that move unnaturally or contain artifacts; essential video objects partially cropped or obscured.

***Excludes:*** Awkward or excessive transitions (B5), abrupt beginnings or endings of video or sections (B5), narrators whose mouths do not match words (C2), off-topic graphics or animations (C3), meaningless or severely distorted still images (C4), inaccurately or illegibly-rendered text (C5).

**B2. Poor audio quality:** Audio, other than spoken language, is noisy or at an inappropriate volume or speed or contains artifacts.

***Includes:*** Very loud or soft volume; sounds played at incorrect speed; noise, distortions, or conversion artifacts or errors.

***Excludes****:* Unnatural or inappropriate features of narration (B4), poor audio transitions or editing (B5), mismatch of audio to video (C2).

**B3. Poor grammar and vocabulary:** Language contains errors of grammar or usage; vocabulary is repetitive or limited.

***Includes:*** Errors of grammar (e.g., word order, verb conjugation, or subject-predicate agreement); repetitive use of words; vocabulary at inappropriate level for subject.

***Excludes:*** Mispronunciations of spoken words (B4), incorrect definitions (A1), subject presented at too basic a level (A4), overdescriptive language (C1).

**B4. Speech/narration irregularities:** Narration or spoken language is unnatural in terms of tone, pace, pronunciation, emotion, or emphasis.

***Includes****:* “Robotic” narration, mispronounced key terms or common words, inappropriate pace or cadence, misplaced stress or emphasis, abbreviations read out as words, excessive or inappropriate emotion, speech at inappropriate speed or volume.

***Excludes:*** Errors in sounds other than spoken language (B2), grammatical errors or poor choice of language (B3), spoken language does not match text or visual elements (C2), script is arranged illogically (C6).

**B5. Poor editing or sequencing:** Audio and/or visual elements are arranged and transitioned in a confusing or distracting manner; applies specifically to audiovisual elements and not to subject matter of video.

***Includes:*** Excessive or inappropriate transitions, unsynchronized audio and visual transitions, inappropriate speed of video, abrupt beginning or ending of video.

***Excludes:*** Animation distortions or artifacts (B1), audio distortions (B2), speech irregularities (B4), mismatch of intentionally-displayed audio and visual elements (C2), illogical flow of concepts or sequence of subject matter (C6).

**Group C: Content – structure/language codes**

**C1. Problematic descriptiveness:** Overdescriptive or nonselectively-descriptive style leading to cluttered, confusing, or unfocused coverage of subject or descriptions of terms or concepts.

***Includes:*** Excessive use of descriptive words, idioms, or cliches; verbose descriptive prose; elaborate yet vague style; descriptions of important and ancillary concepts given equal weight; use of correct analogies where none is necessary.

***Excludes:*** Overgeneralized descriptions (A3), inappropriate level of language for audience (A4), inappropriate or misleading analogies (A5), nonstandard grammar or vocabulary (B3), inappropriately emotional narration (B4).

**C2. Mismatching audio-visual elements:** Disagreement or inconsistency between synchronously-displayed graphics or animations and sounds or narration related to subject.

***Includes:*** On-topic but irrelevant graphics or text; mismatch between spoken descriptions and highlighted parts of graphics; visual and audio channels describing different aspects of subject; mismatch of narrator and narration.

***Excludes:*** Poor-quality animations (B1), inappropriate speech (B4), poorly-executed transitions (B5), off-topic audio or graphic elements (C3), meaningless graphics (C4).

**C3. Distracting or off-topic material:** Presence of audio or visual elements that are unrelated to and unnecessary for the presentation of the subject.

***Includes:*** Music, unnecessary text duplications of spoken words, unnecessary sound effects or filters, graphics or overlays (e.g., watermarks) that obscure on-topic visuals.

***Excludes:*** Unclear or poorly-rendered objects (B1), mismatching on-topic audio and visual elements (C2), unnecessary digressions in script (C6).

**C4. Meaningless graphics:** Nonphysical or garbled images or diagrams relating to subject matter.

***Includes:*** Depictions of fantastical or imaginary objects, distorted or obviously nonphysical depictions of real objects, uninterpretable diagrams or charts.

***Excludes:*** Incorrect descriptions of real objects (A1), unclear, poorly-rendered, or artifact-laden graphics or animations (B1), off-topic visual elements (C3), garbled renderings of text (C5).

**C5. Text irregularities:** Text is rendered in a manner that is illegible or unclear, or has malformed letters or pseudoglyphs.

***Includes*:** Nonsense text, text too small or blurred to be read.

***Excludes*:** Irregular renderings of objects or still images (C4), nongrammatical text (B3), text unrelated to subject matter (C3).

**C6. Disorganization:** Subject matter and its supporting audiovisual scaffolding are presented in an unclear or illogical order with poor linkage of topics and/or no coherent message.

***Includes:*** Confusing or illogical sequence of subtopics, lack of linking or integration between subtopics, unnatural flow from one topic to next, conflicting or incoherent conclusions or messaging of sub-sections.

***Excludes****:* Missing definitions or descriptions (A2), unclear learning objectives or intended level of knowledge (A4), non-synchronized or illogical transitions (B5), confusing or meandering descriptions (C1), mismatches of audio and visual presentations of subject (C2).

|  | **Days online** | **Duration, sec** | **View count** | **Like count** | **Comment count** | **View rate (views/d)** | **Like rate (likes/d)** | **Comment rate (comment/d)** |
| --- | --- | --- | --- | --- | --- | --- | --- | --- |
| **All videos** | 1570 (7 – 6532) | 549 (3 – 6192) | 1.87x10^5^ (2 – 3.16x10^7^) | 3092 (0 – 5.21x10^5^) | 77.7 (0 – 1.23x10^4^) | 89.4 (0.012 – 8673) | 1.84 (0 – 236.4) | 0.042 (0 – 3.39) |
| **Slop** | 266 (8 – 777)** | 69.4 (10 – 586) | 3054 (2 – 7.67x10^4^) | 85.1 (0 – 1800) | 1.6 (0 – 42) | 7.1 (0.041 – 117) | 0.19 (0 – 2.75) | 0.0044 (0 – 0.064) |

**Table S1:** Descriptive statistics for YouTube videos (all videos, n = 814) and YouTube videos designated as slop (slop, n = 47).  Values given are mean with range in parentheses.  ***p* < 0.01 compared to all videos.

|  | **Days online** | **Duration, sec** | **View count** | **Collect count** | **Comment count** | **Share count** | **View rate (views/d)** | **Collect rate (collects/d)** | **Comment rate (comment/d)** | **Share rate (shares/d)** |
| --- | --- | --- | --- | --- | --- | --- | --- | --- | --- | --- |
| **All videos** | 721 (12 – 1960) | 83.7 (5 – 2055) | 7.41x10^4^ (0 – 2.60x10^6^) | 593 (0 – 1.50x10^4^) | 59.7 (0 – 6319) | 482 (0 – 5.47x10^4^) | 1020 (0 – 2.0x10^5^) | 6.21 (0 – 1153) | 0.73 (0 – 158) | 17.2 (0 – 4208) |
| **Slop** | 302 (12 – 686)* | 227 (6 – 1486) | 1.53x10^5^ (81 – 1.4x10^6^) | 708 (0 – 5879) | 102 (0 – 976) | 362 (0 – 2226) | 406 (0.55 – 2794) | 1.89 (0 – 11.7) | 0.30 (0 – 1.95) | 1.41 (0 – 9.39) |

**Table S2:** Descriptive statistics for TikTok videos (all videos, n = 268) and TikTok videos designated as slop (slop, n = 10). Values given are mean with range in parentheses. **p* < 0.05 compared to all videos.
